# 

**Published:** 1845-06

**Authors:** 


### TO READERS, CORRESPONDENTS, &c.

We have received in exchange, the following journals, viz:

The Boston Medical and Surgical Journal for May, 4 nos.

The Western Lancet, May No.

The Southern Medical and Surgical Journal, May.

The Medical News and Library, "

The Western Journal of Medicine and Surgery, "

The New Orleans Medical Journal, "

The Medical Examiner.

The American Journal and Library of Dental Science, March.

Also, the Prospectus of a new Journal about to be issued at Buffalo, N. Y., to be edited by our late colleague, Prof. Flint. It is to be issued monthly; of 20 pages. Dr. Flint possesses high qualifications as a writer, and we heartily wish him success in his undertaking.

